# Prioritizing candidate diseases-related metabolites based on literature and functional similarity

**DOI:** 10.1186/s12859-019-3127-4

**Published:** 2019-11-25

**Authors:** Yongtian Wang, Liran Juan, Jiajie Peng, Tianyi Zang, Yadong Wang

**Affiliations:** 1 School of Computer Science and Technology, Harbin Institute of Technology, Harbin, 150001 People’s Republic of China; 2 School of Life Science and Technology, Harbin Institute of Technology, Harbin, 150001 People’s Republic of China; 3 School of Computer Science, Northwestern Polytechnical University, Xi’an, People’s Republic of China

**Keywords:** Metabolite network, Collaborative filtering, Similarity of metabolites, Random walking with restart

## Abstract

**Background:**

As the terminal products of cellular regulatory process, functional related metabolites have a close relationship with complex diseases, and are often associated with the same or similar diseases. Therefore, identification of disease related metabolites play a critical role in understanding comprehensively pathogenesis of disease, aiming at improving the clinical medicine. Considering that a large number of metabolic markers of diseases need to be explored, we propose a computational model to identify potential disease-related metabolites based on functional relationships and scores of referred literatures between metabolites. First, obtaining associations between metabolites and diseases from the Human Metabolome database, we calculate the similarities of metabolites based on modified recommendation strategy of collaborative filtering utilizing the similarities between diseases. Next, a disease-associated metabolite network (DMN) is built with similarities between metabolites as weight. To improve the ability of identifying disease-related metabolites, we introduce scores of text mining from the existing database of chemicals and proteins into DMN and build a new disease-associated metabolite network (FLDMN) by fusing functional associations and scores of literatures. Finally, we utilize random walking with restart (RWR) in this network to predict candidate metabolites related to diseases.

**Results:**

We construct the disease-associated metabolite network and its improved network (FLDMN) with 245 diseases, 587 metabolites and 28,715 disease-metabolite associations. Subsequently, we extract training sets and testing sets from two different versions of the Human Metabolome database and assess the performance of DMN and FLDMN on 19 diseases, respectively. As a result, the average AUC (area under the receiver operating characteristic curve) of DMN is 64.35%. As a further improved network, FLDMN is proven to be successful in predicting potential metabolic signatures for 19 diseases with an average AUC value of 76.03%.

**Conclusion:**

In this paper, a computational model is proposed for exploring metabolite-disease pairs and has good performance in predicting potential metabolites related to diseases through adequate validation. This result suggests that integrating literature and functional associations can be an effective way to construct disease associated metabolite network for prioritizing candidate diseases-related metabolites.

## Background

While a gene-based approach has contributed to our knowledge on the genomic space of possible genes and proteins [1–4], it is increasingly understood that such an approach is far from sufficient because most cellular components work intricate networks of regulatory, metabolic, and protein interactions [5–8]. As the end products of cellular regulatory processes, metabolites can be considered as the ultimate response of biological systems to genetic or environmental changes [9]. In biological systems, metabolomics, which is an emerging area of research, can not only contribute to the discovery of metabolic signatures for disease diagnosis, but is very helpful to illustrate the underlying molecular disease-causing mechanisms [9–12]. Furthermore, metabolites are easier to be analyzed for recognizing diseases at the molecular level compared to genes, mRNA transcripts and proteins related to diseases, among which there are large quantities of intricate interactions. Therefore, metabolisms, as the final products of cellular regulatory processes, can be a significant factor to illustrate the disease-causing mechanisms.

Nowadays, the advanced technology is really helpful to researchers for studying diseases in the molecular level [13–17]. And more researchers have devoted their work to metabolomics for revealing more information about diseases. Breitling, R et al. [18] utilized Fourier transform mass spectrometry data to make prediction of metabolic networks. In 2010, Gao, J et al. [19] developed a plugin for visualizing and interpreting metabolomic data in human metabolic networks. Considering the global importance of metabolites and the unique character of metabolomic profile, Li Feng et al. [20] proposed a network-based method for metabolite pathway identification. In 2016, Sergushichev, AA et al. [21] presented a web-service for integrated transcriptional and metabolic network analysis, focusing on identification of the most changing metabolic subnetworks between two conditions of interest. Wang et al. [22] identified potential urinary biomarkers for early colorectal cancer detection utilizing NMR-based metabolomic techniques. Recently, Ohtana, Yuki et al. [23] made analysis of drug-endogenous human metabolite similarities and 3D-Structure similarity based network of Secondary Metabolites [24]. To figure out whether metabolite networks are reproducible across different populations, Iqbal, Khalid et al. [25] investigated similarity of metabolite networks in four German population-based studies (EPIC-Potsdam, EPIC-Heidelberg, KORA and CARLA). From the above it can be seen that researchers are paying more attention to metabolite research and metabolomics has developed rapidly.

As the link between genotypes and phenotypes, one metabolite is not always related to a sole disease, and the impact of certain disease spreads among functionally related metabolites in a network [26]. Thus, adjacent metabolites with functional associations in this network tend to relate to the same diseases or similar ones [6]. This suggests that the functional associations between metabolites can be measured by the similarities of diseases. Therefore, we aimed to identify more disease-related metabolites by analysis the metabolite and disease data.

Now there have been many methods to calculate medical terminology similarity [27–30]. But to our knowledge, no methods had been proposed to compute metabolite similarity based on collaborative filtering (CF) [31] with the functional similarities between diseases as weight. CF can effectively utilize associations among other similar members and discover potential but not yet found interests. It is able to finish personalized recommendation with high degree of automation. Thus, a disease associated metabolite network (DMN) can be built based on modified collaborative filtering, which takes advantage of the entire interaction network. However, relying entirely on metabolite-related diseases greatly limits the utility of the method because many metabolites still have very few or no associated diseases. To overcome this limitation, a new disease-associated metabolite network (FLDMN) is built by fusing functional associations and scores of literatures from STITCH database [32]. Finally, FLDMN is utilized to identify potential disease-related metabolites based on network random walk.

## Materials and methods

**Fig. 1:**
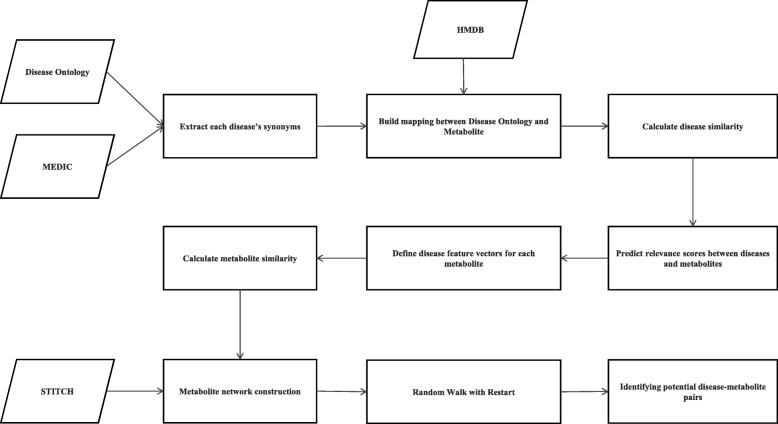
The flow chart of building FLDMN to identify potential disease-related metabolites

To clarify the research that we do, the workflow of the computational model is shown in Fig. 1. First, we integrate information from Human Disease Ontology (DO) [33], Merged Disease vocabulary (MEDIC) [34] and Human Metabolome Database (HMDB) [35] to establish mapping between DO terms and metabolites. Next, we define a feature vector for each metabolite to calculate the similarities between metabolites. Given the associations between metabolites and diseases, disease functional similarities are calculated by FNSemSim [29] and added to the dimensions of this vector as relevance scores between diseases and metabolites. Based on functional associations between metabolites, a disease-related metabolite network (DMN) is built. Subsequently, extracting scores of literatures from STITCH, we build a new network of disease related metabolites (FLDMN). Random Walking with Restart (RWR) is applied in this new network to output the ranking of candidate disease-related metabolites. Therefore, the potential relationships between diseases and metabolites can be identified.

### Data collection

#### Disease database

Merged Disease vocabulary (MEDIC) [34] from Comparative Toxicogenomics Database (CTD) [36] is a modified subset of descriptors from “Diseases” category of Medical Subject Headings (MeSH) [37]. MEDIC is used to curate gene–disease and chemical–disease associations in CTD. In this study, we will use the “Synonyms” field and the “DiseaseName” field of MEDIC as a part of a combined vocabulary for mapping.

The Human Disease Ontology (DO) [33] is a community driven standards-based ontology that is focused on representing common and rare disease concept, which provides researchers with an open source ontology for the integration of biomedical data that is associated with human disease. The content of each disease in DO is a node, which has a parent-child relationship with others. All of these nodes are organized in a directed acyclic graph (DAG) with an ‘IS_A’ relationship. In this study, terms of DO will also be utilized as a part of a combined vocabulary. Finally, we use this combined vocabulary to annotate DO with metabolite-related diseases.

#### Human metabolome database

The Human Metabolome Database (HMDB) [35] is a freely metabolome database with detailed information about small molecule metabolites in the human body. Currently, HMDB involves 11,400 metabolites, which contains 835 disease associated metabolites with 825 diseases. In this study, we will use two different versions of HMDB, which released in Dec. 2017 and Apr. 2018, for constructing the metabolite network and extracting testing sets, respectively.

STITCH [32] is a database of known and predicted interactions between chemicals and proteins, which interactions includes direct and indirect associations. Currently, STITCH database contains 9,643,763 proteins from 2031 organisms. In this study, eligible interactions from STITCH are involved in the reconstruction of metabolic network.

### Methods of the metabolite network construction

#### Mappings between diseases and metabolites

The xml file which contains information about metabolites can be found in HMDB web site. However, we find that these diseases in HMDB don’t have any mapping with DO terms when this file is parsed. Therefore, we build mappings between DO terms and diseases in HMDB. As comprehensive disease corpuses, MEDIC and DO both contain abundant disease terms. First, we parse the HMDB file to get disease-related metabolites. Then we annotate DO entries with the terms from MEDIC and create a combined vocabulary of disease terms. Finally, mappings between DO terms and diseases in HMDB are built reference to this combined vocabulary.

#### Metabolite similarity calculation based on modified collaborative filtering

**Fig. 2:**
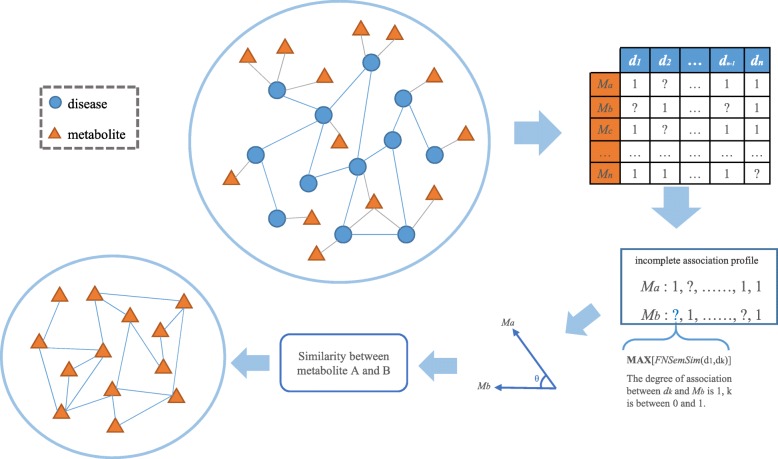
The workflow of calculating metabolite similarity

As one of the most successful technologies for recommender systems [38], collaborative filtering has been developed and improved over the past decade. In this study, we define associations between metabolites based on modified collaborative filtering. In order to achieve this, each metabolite can be seen as a vector, which dimension is defined as the number of diseases. Through mapping diseases to metabolites, we obtain a set of initial vectors. Disease similarities are employed to predict the score of one dimension in a vector when there is no score in this dimension. Finally, we calculate similarities between vectors by cosine measure. The workflow for calculating metabolite similarity is shown in Fig. 2.

##### Metabolite-related diseases similarity

After obtaining metabolite-related diseases, we calculate similarities between these diseases as inputs of further predicting relevance scores. In this paper, the method named FNSemSim [29], which we previously developed, is utilized to calculate disease similarities. This method measures the similarity of diseases by a fused gene functional network of HumanNet [39] and FunCoup [40]. Through assessment the method has good performance for calculating similarities between diseases.

Let a pair of gene sets *G*_*a*_ = {*g*_*a*1_, *g*_*a*2_, …} and *G*_*b*_ = {*g*_*b*1_, *g*_*b*2_, …} be related to disease *d*_*a*_ and *d*_*b*_, respectively. The similarity between disease *d*_*a*_ and *d*_*b*_ is defined as follows:

1 $$ {\displaystyle \begin{array}{c} DiseaseFunSim\left({G}_a,{G}_b\right)=\frac{\sum \limits_{1\le i\le num\left({G}_a\right)}{R}_{G_b}\left({g}_{ai}\right)+\sum \limits_{1\le j\le num\left({G}_b\right)}{R}_{G_a}\left({g}_{bj}\right)}{\left|{G}_a\right|+\left|{G}_b\right|}\\ {}{g}_{ai}\in {G}_a,{g}_{bj}\in {G}_b\end{array}} $$

where |*G*_*a*_| and |*G*_*b*_| respectively represents the numbers of genes related to disease *d*_*a*_ and *d*_*b*_; and *R*_*G*_(*g*) represents the connection weights in the fused functional association network (see details in [29]). Finally, FNSemSim could be defined as follows:

2 $$ FNSemSim\left({d}_a,{d}_b\right)= DiseaseFunSim\left({G}_a,{G}_b\right)\ast \frac{\left|{G}_a\right|\left|{G}_b\right|}{\left|{G}_{MICA}\right|\left|{G}_{MICA}\right|} $$

where |*G*_*a*_| and |*G*_*b*_| represent the size of two gene sets, *G*_*a*_ and *G*_*b*_, related to disease *d*_*a*_ and *d*_*b*_ in Disease Ontology, respectively; |*G*_*MICA*_| represents the number of genes related to the most informative common ancestor of *d*_*a*_ and *d*_*b*_. Finally, we normalize similarities between pair-wised diseases associated with metabolites.

##### Relevance scores between diseases and metabolites

We utilize the similarities between diseases associated with metabolites to predict the relevance score of a disease that is not directly related to one metabolite. We define *M* and *D* as the set of metabolites and the set of related diseases, respectively. *DR*_*m*_ is defined as the set of diseases directly related to metabolite *m*. The predicted association score between disease *d* and metabolite *m* is defined as follows:

3 $$ PA\left(d,m\right)=\left\{\begin{array}{c}\mathit{\operatorname{MAX}}\left( FNSemSim\left( di,d\right)\right)\\ {} 1\end{array}\kern1.12em \begin{array}{l} di\in DRm\kern0.5em and\kern0.5em d\notin DRm\\ {}d\in DRm\end{array}\right. $$

where *m* ∈ *M*, *d* ∈ *D*, *DRm* ⊆ *D* and 1 ≤ *i* ≤ |*DRm*|; here, |*DRm*| represents the number of diseases in the set of *DR*_*m*_. We define a vector of each metabolite with |*D*| dimension, respectively. |*D*| represents the size of the disease set *D*. For each metabolite we can define its vector $\overrightarrow{m}$ as follows:

4 $$ {\displaystyle \begin{array}{c}\overrightarrow{m}=\left( PA\left(d 1,m\right)\kern0.5em ,\kern0.5em \cdots \cdots, \kern0.5em PA\left( dk,m\right)\right)\\ {}m\in M,\kern0.5em 1\le k\le \left|D\right|\end{array}} $$

where |*D*| represents the size of the disease set *D*; $\overrightarrow{m}$ represents the score vector of metabolite *m*; and *PA*(*d*k, *m*) is the score between disease *d*_*k*_ and metabolite *m*. Now, we can obtain |*M*| vectors of metabolites related to diseases.

##### Metabolite similarity

Because each metabolite can be depicted by a multi-dimensional vector, we can find associations between metabolites in multi-dimensional space that is composed of metabolite-related diseases. Thus, we use cosine measure to calculate the similarity between any two vectors of metabolites. The association between metabolite *m*_*1*_ and metabolite *m*_*2*_ is defined as follows:

5 $$ \mathrm{DMN}\left({\mathrm{m}}_1,{\mathrm{m}}_2\right)=\frac{\sum_1^{\mathrm{n}}\left({PA}_{1,i}\times {PA}_{2,\mathrm{i}}\right)}{\sqrt{\sum_1^{\mathrm{n}}{\mathrm{PA}}_{1,i}^2}\times \sqrt{\sum_1^{\mathrm{n}}{\mathrm{PA}}_{2,i}^2}} $$

where *PA*_*k,i*_ represents the association score between metabolite *m*_*k*_ and disease *d*_*i*_ in the *i*-th dimension of the vector ${\overrightarrow{m}}_{\mathrm{k}}$. The range of *DMN*(*m*_*1*_, *m*_*2*_) is 0 to 1 because these values in all dimensions are positive numbers. Finally, we obtain all associations of pair-wised metabolites related to diseases and build a disease-associated metabolite network (DMN).

##### Metabolite network reconstruction

In DMN, there exist only functional associations between disease-related metabolites, because all the links in this network are created by taking special phenotype as a measure. In view of this, we take metabolite related literatures as weight and extract text mining scores from STITCH to improve associations between metabolites in DMN. The combined weight of metabolite *m*_*1*_ and metabolite *m*_*2*_ is defined in Eq. 6.

6 $$ \mathrm{FLDMN}\left({m}_1,{m}_2\right)=1\hbox{-} \left(1\hbox{-} DMN\left({m}_1,{m}_2\right)\right)\left(1\hbox{-} ST\left({m}_1,{m}_2\right)\right) $$

where *ST*(*m*_*1*_, *m*_*2*_) represents the text mining score of metabolite *m*_*1*_ and metabolite *m*_*2*_ in STITCH. The ranges of *ST*(*m*_*1*_, *m*_*2*_) and *DMN*(*m*_*1*_, *m*_*2*_) are both 0 to 1. Finally, we utilize the new associations between metabolites to reconstruct the network FLDMN.

### Identifying novel candidate disease-related metabolites

The associations between a metabolite and its first neighbours are shown in FLMDN, but those between it and all the others in this network are ignored. To identify novel candidate disease-related metabolites by fully exploiting the global functional similarities of metabolites in this metabolite network, we employ RWR [41] to make relationship mining between any two metabolites in this network.

As a global optimization method, RWR can output more information about one metabolite and all the others in the network. In a network, the random walker starts from the root node and migrate to neighbouring nodes with the probabilities from that node to the others. After several iterations, the probabilities from the root node to all the other nodes in this network will become stable. Because RWR is a popular method based on graph structure, we do not repeat it here (see [41] for RWR details). Finally, we can obtain a rank for each metabolite in this network by RWR.

## Results

### Metabolites and diseases

We extracted 8704 disease terms from Disease Ontology (released in Nov. 2017) and calculated similarities between them. Those pair-wised diseases whose similarities are zero removed, there remain 3,801,586 associations among 4703 disease terms. Meanwhile, we found 1406 relationships between diseases and metabolites when DO terms are mapped to the diseases in HMDB (released in Dec. 2017) referring to the combined vocabulary of DO and MEDIC. Two hundred forty-eight diseases and 600 metabolites are totally contained.

**Fig. 3:**
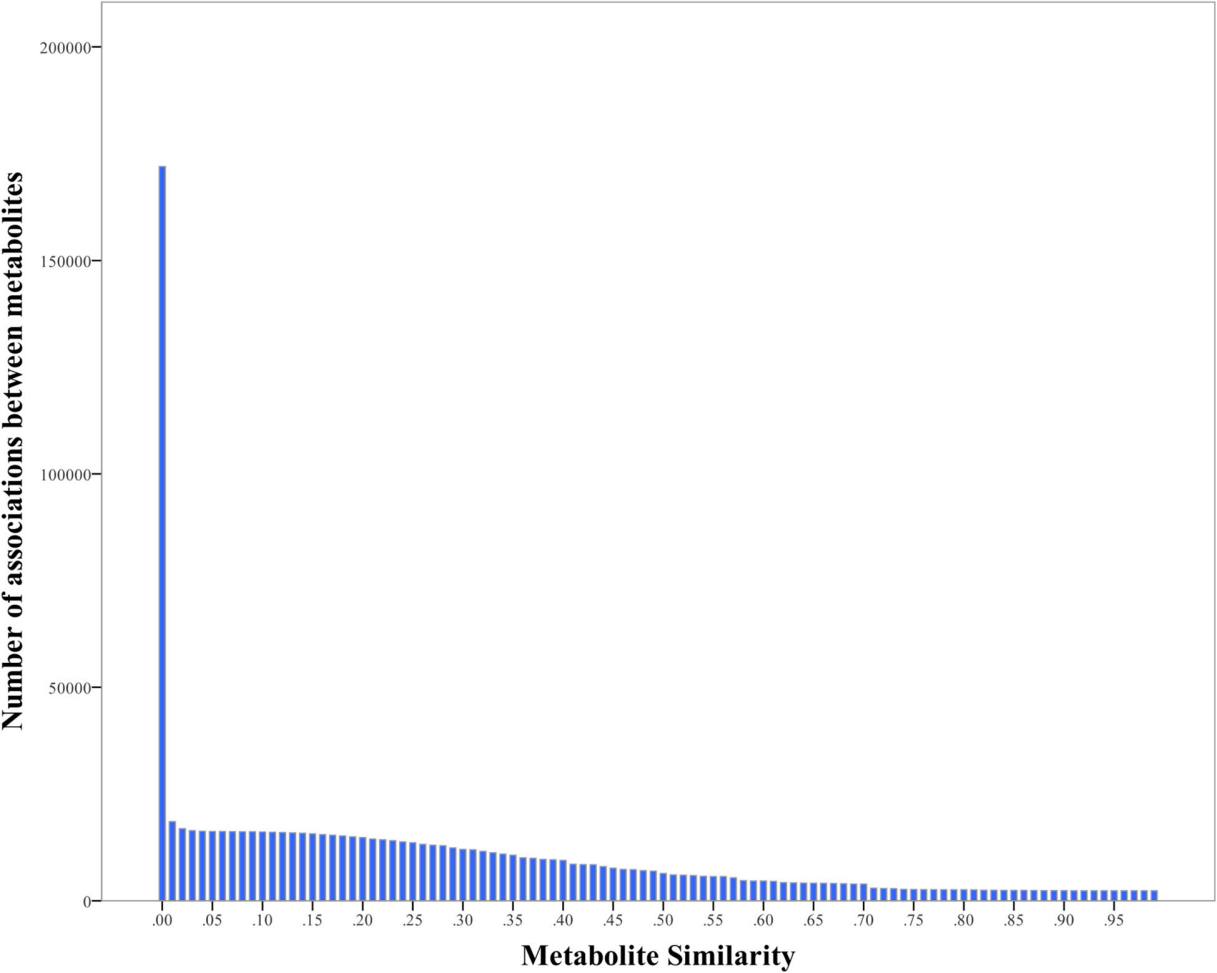
The quantities distribution of metabolite associations with different similarities

We calculated 197,700 similarities among these 600 metabolites, and found that there were a large number of very weak associations and 25,709 irrelevant pair-wised metabolites in these results. To reduce the influence of noise on the network, we analyzed the distribution of metabolite associations with different similarities as thresholds. As we can see in Fig. 3, the number of associations is declining while the threshold is increasing. And when the threshold approaches 0.01, the alternation in the number of associations levels off. Therefore, we filtered out 153,455 associations with 0.01 as a threshold. Finally, we built the network DMN with 18,536 associations among 587 metabolites that are associated with 245 diseases.

To improve associations between metabolites in DMN, we extracted text mining scores of pair-wised metabolites from STITCH. We obtained a network named ST_SUBNET composed of 28,292 associations among 485 metabolites from STITCH. Finally, FLDMN is built and contains 28,715 associations among 587 metabolites associated with 245 diseases.

### Performance

To assess the performance of DMN and FLDMN, we performed a validation with 78 known disease metabolites associated with 19 diseases obtained from HMDB (released in Apr. 2018). But these known disease-related metabolites had no association with these 19 diseases in HMDB (released in Dec. 2017). The detailed statistics for evaluating disease-related metabolite networks are given in Table 1. For each disease, all of tested metabolites, which exist in the version of both 2017 and 2018 like other metabolites involving in the performance evaluation, only have associations with this disease in the version of 2018.

**Table 1:** Statistics for evaluating disease-related metabolite network

Disease name	Disease Ontology	Test node	Positive group	Version 2017	Version 2018
L-2-hydroxyglutaric aciduria	DOID:0050574	2	4	2	4
medium chain acyl-CoA dehydrogenase deficiency	DOID:0080153	1	16	15	16
short chain acyl-CoA dehydrogenase deficiency	DOID:0080154	1	4	3	4
Crohn’s colitis	DOID:0060192	5	8	3	16
cerebrotendinous xanthomatosis	DOID:4810	5	7	2	9
maple syrup urine disease	DOID:9269	6	23	17	24
abetalipoproteinemia	DOID:1386	3	4	1	4
celiac disease	DOID:10608	11	22	11	82
methylmalonic acidemia	DOID:14749	1	2	1	2
irritable bowel syndrome	DOID:9778	5	7	2	15
Fanconi syndrome	DOID:1062	1	2	1	5
citrullinemia	DOID:9273	6	8	2	8
inflammatory bowel disease 1	DOID:0110892	5	8	3	16
isovaleric acidemia	DOID:14753	2	11	9	12
type 2 diabetes mellitus	DOID:9352	1	27	27	27
aromatic L-amino acid decarboxylase deficiency	DOID:0090123	2	9	7	12
cholesterol ester storage disease	DOID:14502	1	2	1	2
congenital adrenal hyperplasia	DOID:0050811	15	18	3	28
Crohn’s disease	DOID:8778	5	8	3	16

**Fig. 4:**
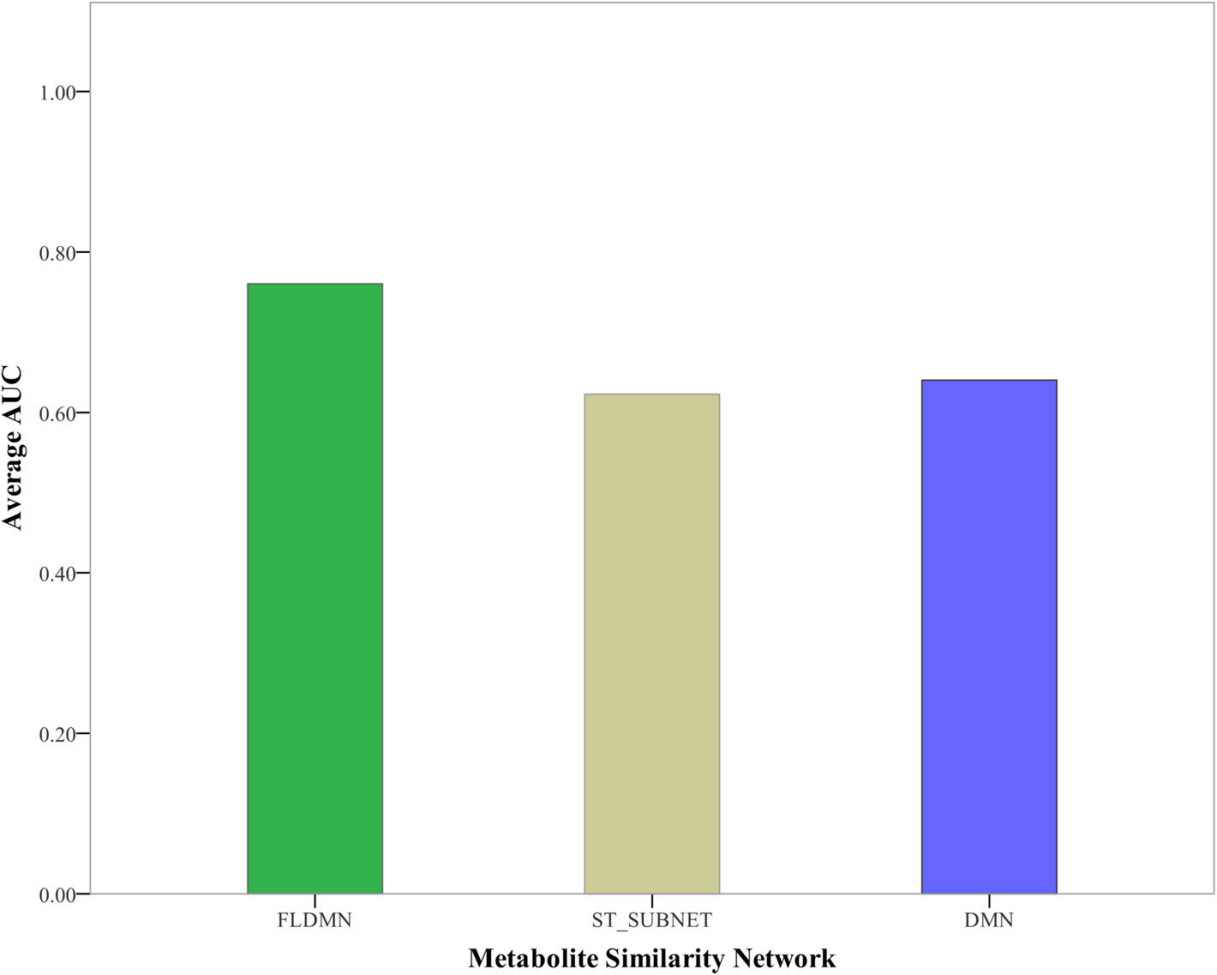
Average AUC of three metabolite networks. The average AUC of FLDMN reaches 76.03%, while the average AUC of ST_SUBNET is 62.3% and DMN has an average AUC value of 76.03%

As a result, the average AUC (area under the receiver operating characteristic curve) of DMN for 19 diseases reached 64.35%. And FLDMN was proved to be successful in predicting novel metabolic signatures for 19 diseases with an average AUC value of 76.03%. Meanwhile, we also assessed ST_SUBNET to figure out whether the excellent performance of FLDMN is only due to ST_SUBNET. As shown in Fig. 4, the average AUC of ST_SUBNET reached 62.3% that was a little lower than DMN. This illustrates that the performance of FLDMN is the combined effect of DMN and ST_SUBNET. But AUC of ST_SUBNET doesn’t mean that STITCH has an average performance because ST_SUBNET is only a small part of its.

**Fig. 5:**
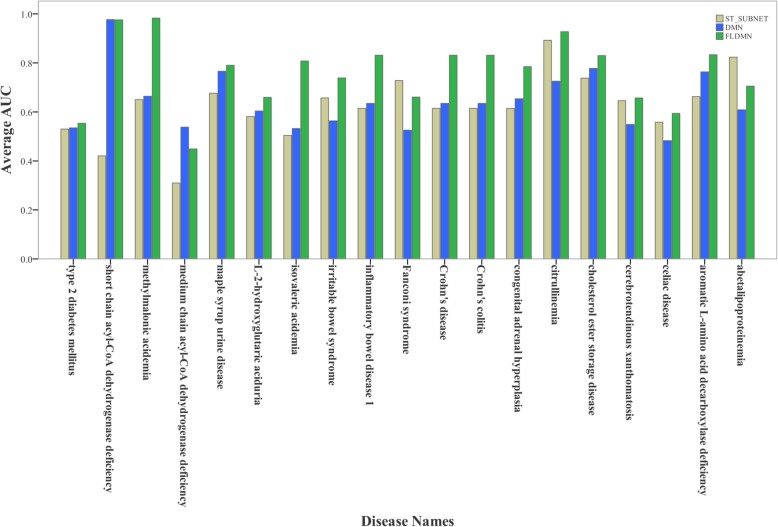
Performances of three metabolite networks to predict candidate metabolites related with a given disease. ST_SUBNET, DMN and FLDMN are utilized to predict candidate metabolites for each of these 19 diseases, respectively. For a given disease, the three bars with different colours represent average AUC of the three metabolite networks, correspondingly

We found that our method had outstanding performance on some diseases. For example, short chain acyl-CoA dehydrogenase deficiency (DOID:0080154) had an AUC of 97.68% in DMN, and there were 9 diseases whose AUC were more than 80% in FLDMN, as shown in Fig. 5. Among these 19 diseases, AUC of celiac disease (DOID:10608) in DMN was 48.1% while it reached 59.4% in FLDMN. As we can see in Table 1, the number of metabolites associated with celiac disease in HMDB (2018) was 71 more than that in 2017 version, while there were 22 positive samples for 11 test nodes. It implies that the relatively small number of positive samples could affect the result of predicting candidate metabolites related with celiac disease. In addition, the AUC of medium chain acyl-CoA dehydrogenase deficiency (DOID:0080153) was smaller in FLDMN than in DMN. Part of it may be the fact that some noise is introduced by ST_SUBNET. But in general, the performance of FLDMN is outstanding in predicting candidate disease-related metabolites.

### Case study

We used Alzheimer’s disease (DOID:10652) as one of case studies to further evaluate the performance of our computational model in predicting potential disease-related metabolites. First, we utilized the metabolite data from HMDB (released in Apr. 2018) to build FLDMN. We employed RWR and found that S-Adenosylhomocysteine (HMDB0000939) had a high score of 0.91 for Alzheimer’s disease, which was ranked in top 3%. But the relationship between S-Adenosylhomocysteine and Alzheimer’s disease was not included in HMDB (released in Apr. 2018). S-Adenosylhomocysteine has been demonstrated to be related to Alzheimer’s disease [42]. L-Cysteine (HMDB0000574) was ranked in top 5% for Alzheimer’s disease, which was a naturally occurring, sulfur-containingamino acid. It has been reported as a potentially metabolic intermediary of Alzheimer’s disease [43]. Substance P (HMDB0001897), an 11-amino acid neuropeptide, was ranked in top 10% for Alzheimer’s disease. Rosler, N et al. [44] have found that AD patients with late disease onset showed significantly higher values of Substance P than early onset patients. We also found potential metabolites related to leukemia (DOID:1240). Putrescine (HMDB0001414), N8-Acetylspermidine (HMDB0002189) and N1-Acetylspermidine (HMDB0001276) were ranked in top 5% for leukemia, which were well documented to be associated with leukemia [45]. Type 1 diabetes mellitus (DOID:9744) is characterized by loss of the insulin-producing beta cells of the pancreatic islets, leading to insulin deficiency. We also applied this method to type 1 diabetes mellitus to find potential some metabolites. Pyruvic acid (HMDB0000243), 3-Hydroxyisovaleric acid (HMDB0000754), Dimethylamine (HMDB0000087) and Citric acid (HMDB0000094) were ranked in top 20% for type 1 diabetes mellitus, and these metabolites were reported in the study of type 1 diabetes mellitus [46].

## Discussion

**Fig. 6:**
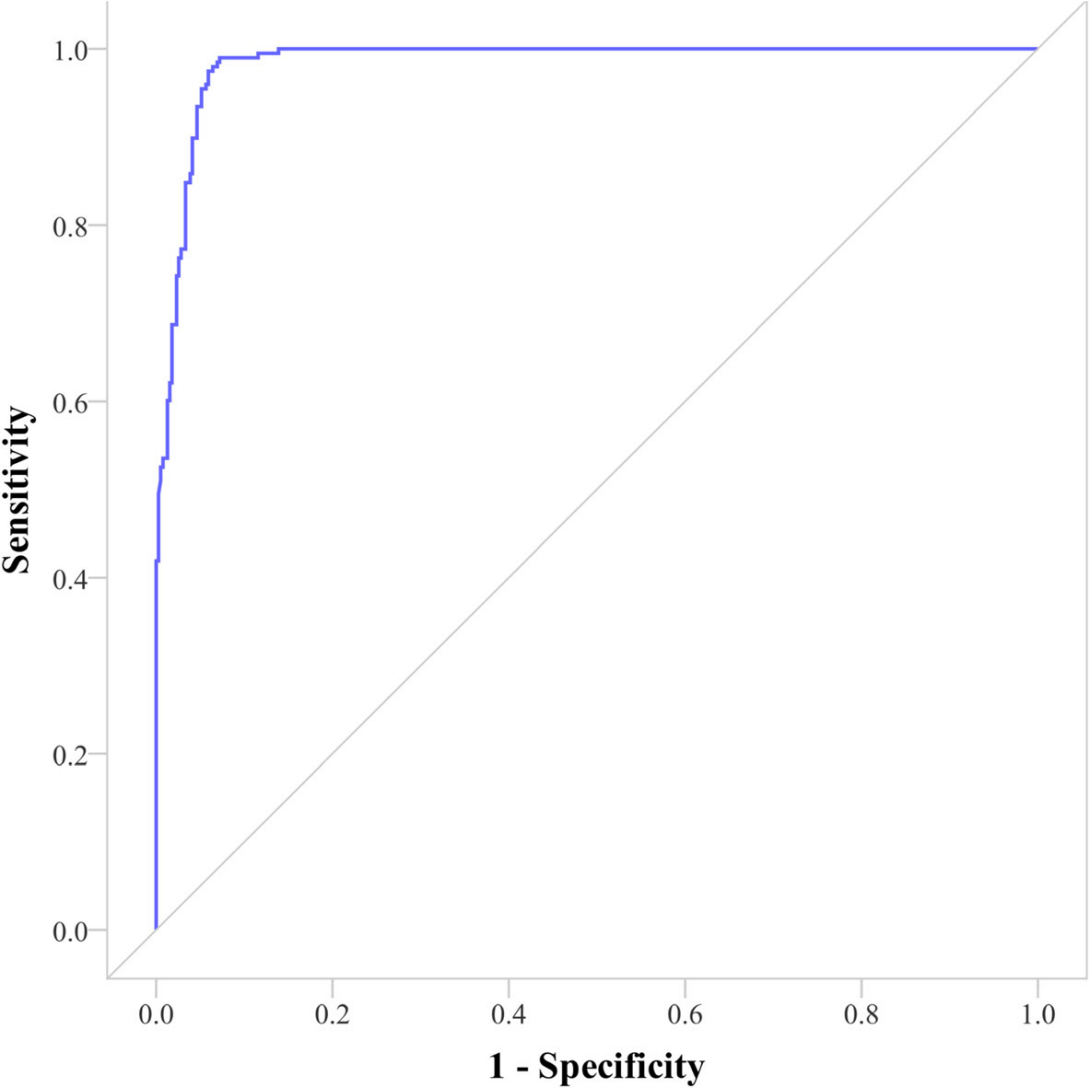
ROC of L-Threonine based on FLDMN

We identified candidate metabolites related with a certain disease in FLDMN and used AUC to measure its performance. We can also use FLDMN to prioritizing candidate disease-related metabolites for a certain metabolite. The AUC will be better. Because most of metabolites often associate with more than one diseases, positive samples will get more for a certain metabolite. Therefore, they can be tested first in the rank for this metabolite in FLDMN. Take L-Threonine (HMDB0000167) as an example. There are totally seven metabolites that have disease-related associations with it. As we can see in Fig. 6, its AUC was 98.44%. Subsequently, we used these above-mentioned test nodes as a target node respectively to rank candidate disease-related metabolites. The average AUC was 89.88%.

**Fig. 7:**
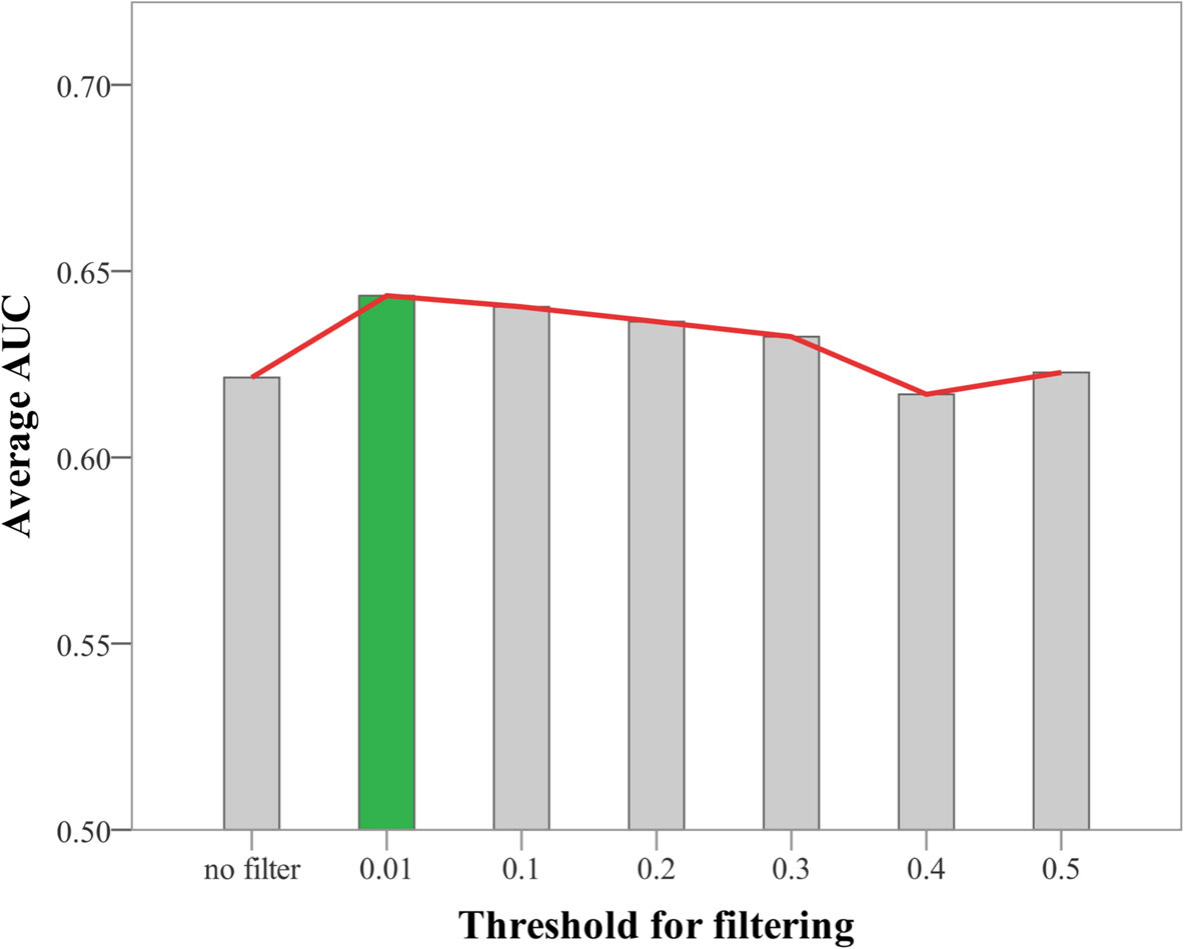
Average AUC of DMN with different thresholds

When DMN was built, the threshold was set as 0.01 to filter weak links. We did some experiments later to figure out whether the threshold was reasonable. There were seven networks with different thresholds to be constructed, respectively. As shown in Fig. 7, the average AUC of DMN with 0.01 as a threshold was outstanding. Therefore, there will be better results to use 0.01 as a threshold.

## Conclusions

As the link between genotypes and phenotypes, metabolites can be used to explain the underlying molecular disease-causing mechanisms. For this purpose, we proposed a computational model to build a disease-related metabolite network and identified candidate metabolites related to diseases.

First, we used FNSemSim to calculate similarities of pair-wised diseases. Subsequently, we defined associations between metabolites by modified collaborative filtering and built a disease associated metabolite network (DMN). To improve these associations, a new disease associated metabolite network by fusing functional associations and scores of literatures (FLDMN) was constructed. Finally, we used RWR to prioritize candidate disease-related metabolites.

The results showed that our method was proved to be successful in predicting novel metabolic signatures for 19 diseases with an average AUC value of 76.03%. And it will be helpful for researchers in metabolomics. Take Alzheimer’s disease and leukemia as examples, we found some unknown metabolites that were mapped to these diseases through our network.

## Data Availability

All the datasets used in this paper could be downloaded from websites.

## Abbreviations

AUC: Area under the receiver operating characteristic curve
CF: Collaborative filtering
CTD: Comparative toxicogenomics database
DAG: Directed acyclic graph
DO: Disease ontology
HMDB: Human Metabolome Database
MEDIC: Merged disease vocabulary
MeSH: Medical Subject Headings
RWR: Random walking with restart
STITCH: Search tool for interactions of chemicals
